# The design of a sample rapid magnetic resonance imaging (MRI) acquisition protocol supporting assessment of multiple articular tissues and pathologies in knee osteoarthritis

**DOI:** 10.1016/j.ocarto.2024.100505

**Published:** 2024-07-23

**Authors:** Felix Eckstein, Thula Cannon Walter-Rittel, Akshay S. Chaudhari, Nicholas M. Brisson, Tazio Maleitzke, Georg N. Duda, Anna Wisser, Wolfgang Wirth, Tobias Winkler

**Affiliations:** a Research Program for Musculoskeletal Imaging, Center for Anatomy & Cell Biology, Paracelsus Medical University, Salzburg, Austria; b Ludwig Boltzmann Institute for Arthritis and Rehabilitation (LBIAR), Paracelsus Medical University, Salzburg, Austria; c Chondrometrics GmbH, Freilassing, Germany; d Charité – Universitätsmedizin Berlin, Corporate Member of Freie Universität Berlin and Humboldt-Universität zu Berlin, Department of Radiology, Berlin, Germany; e Department of Radiology, Stanford University, Stanford, CA, USA; f Berlin Institute of Health at Charité – Universitätsmedizin Berlin, Julius Wolff Institute, Berlin, Germany; g Berlin Movement Diagnostics (BeMoveD), Center for Musculoskeletal Surgery, Charité – Universitätsmedizin Berlin, Berlin, Germany; h Charité – Universitätsmedizin Berlin, Corporate Member of Freie Universität Berlin and Humboldt-Universität zu Berlin, Center for Musculoskeletal Surgery, Berlin, Germany; i Trauma Orthopaedic Research Copenhagen Hvidovre (TORCH), Department of Orthopaedic Surgery, Copenhagen University Hospital - Amager and Hvidovre, Hvidovre, Denmark; j Department of Clinical Medicine, University of Copenhagen, Copenhagen, Denmark; k Charité – Universitätsmedizin Berlin, Corporate Member of Freie Universität Berlin, Humboldt-Universität zu Berlin and Berlin Institute of Health, Berlin Institute of Health Center for Regenerative Therapies, Berlin, Germany

**Keywords:** Clinical trial, Osteoarthritis (OA), MRI acquisition protocol, Synovitis, Early- and late-stage disease

## Abstract

**Objective:**

This expert opinion paper proposes a design for a state-of-the-art magnetic resonance image (MRI) acquisition protocol for knee osteoarthritis clinical trials in early and advanced disease. Semi-quantitative and quantitative imaging endpoints are supported, partly amendable to automated analysis. Several (peri-) articular tissues and pathologies are covered, including synovitis.

**Method:**

A PubMed literature search was conducted, with focus on the past 5 years. Further, osteoarthritis imaging experts provided input. Specific MRI sequences, orientations, spatial resolutions and parameter settings were identified to align with study goals. We strived for implementation on standard clinical scanner hardware, with a net acquisition time ≤30 ​min.

**Results:**

Short- and long-term longitudinal MRIs should be obtained at ≥1.5T, if possible without hardware changes during the study. We suggest a series of gradient- and spin-echo-sequences, supporting MOAKS, quantitative analysis of cartilage morphology and T2, and non-contrast-enhanced depiction of synovitis. These sequences should be properly aligned and positioned using localizer images. One of the sequences may be repeated in each participant (re-test), optimally at baseline and follow-up, to estimate within-study precision. All images should be checked for quality and protocol-adherence as soon as possible after acquisition. Alternative approaches are suggested that expand on the structural endpoints presented.

**Conclusions:**

We aim to bridge the gap between technical MRI acquisition guides and the wealth of imaging literature, proposing a balance between image acquisition efficiency (time), safety, and technical/methodological diversity. This approach may entertain scientific innovation on tissue structure and composition assessment in clinical trials on disease modification of knee osteoarthritis.

## 1 Introduction

Studies investigating osteoarthritis (OA) status, progression, or response to intervention frequently employ liquid or imaging biomarkers [1]. The advantage of imaging is that it visualizes articular tissues directly, providing exquisite soft tissue contrast when magnetic resonance imaging (MRI) is used [2]. Commonly in OA, articular tissue pathology is evaluated by expert radiologists using semi-quantitative MRI scoring systems [[3], [4], [5], [6]], but articular tissue properties may also be measured quantitatively using morphometry [[7], [8], [9]]. Another avenue is relaxometry [10,11] that derives MRI-specific relaxation parameters related to biochemical composition of specific tissues. The detection of specific pathological processes is best supported by different MRI sequences with specific parameters, resolution, orientation, etc.

A dedicated MRI acquisition protocol (i.e. a series of sequential acquisitions within one scanning session) must balance the number of MRI sequences used, enabling specific image assessments that answer study questions within the examination time that the patient is able, or willing, to tolerate. Overly long imaging times can be detrimental to image quality, potentially resulting in motion artifacts due to patient physical discomfort and a negative impact on patient experience that may reduce follow-up visit compliance [12]. Further, clinical trial MRI time often competes with scan time reserved for clinical routine imaging. Shorter protocols that have proven useful in clinical trials are more easily translated into clinical practice, and may help standardizing of general medical diagnostics and generating comparable results across different hospitals and countries.

The image acquisition protocol of the Osteoarthritis Initiative (OAI) is well established [13,14], containing fast (intermediate weighted) spin-echo sequences for semi-quantitative tissue evaluation, and a high resolution coronal spoiled gradient echo and sagittal double echo steady state (DESS) [15] for quantification of cartilage morphology [7,8,16]. The DESS also facilitates morphometry of meniscus morphology and extrusion [[17], [18], [19]], and bone size and shape [8,[20], [21], [22]]. A multi-echo-spin-echo (MESE) sequence was finally acquired to measure transverse (spin-spin) relaxation times (T2) of cartilage. T2 is related to the speed by which protons lose phase coherence after excitation, the rate of decay impacted by free water molecules that prolong transverse magnetization [23]. It thus can be employed to estimate articular cartilage matrix composition, specifically hydration and collagen [24,25]. T2 has been correlated with cartilage histological grading [26], mechanical properties [27], and early OA before matrix loss occurs [10,[28], [29], [30]]. The OAI imaging protocol required 58 ​min (39 ​min for the target, and 19 ​min for the secondary knee), not including patient set-up, coil repositioning, and time for pre-scanning. In 2015, a consensus-driven approach was put forward on how imaging may be best applied to knee OA (KOA) trials [31]. The review provided information on acquisition methods, protocol and hardware recommendations, quality control procedures, performance metrics for assessments, and clinical research recommendations [31]. Given concerns about image quality and time constraints, the OAI protocol is not ideal for clinical trials today. New MRI sequences and modifications have been introduced to replace, supplement, or accelerate traditional sequences [2,11,32,33].

The purpose of this expert opinion design paper is to propose, implement, test, and discuss a state-of-the-art image acquisition protocol for KOA clinical trials, applicable in either early or advanced KOA. A 30-min net acquisition was targeted; yet, the protocol supports analysis of multiple desirable semi-quantitative and quantitative assessments, pertinent to a multitude of articular and peri-articular tissues and pathological processes that include synovitis [34,35]. Quantitative assessment should be amendable to automated analysis [[36], [37], [38], [39]]. The choice of MRI sequences and their specific role in studying KOA relevant structural endpoints will be discussed in context of the scientific literature with the aim of helping to bridge the gap between technical manuals (i.e. protocol acquisition guides) for KOA used in clinical studies and the wealth of imaging literature.

## 2 Methods

A design for an MRI protocol was developed for use in two clinical trials within the PeRsOnalized Therapies for Osteoarthritis (PROTO) (Advanced PeRsOnalized Therapies for Osteoarthritis) project (No: 101095635, HORIZON-HLTH-2022-STAYHLTH-02-01). (i) PROTO-PTOA (PTOA ​= ​posttraumatic OA) studies patients with pre-stage KOA (anterior cruciate ligament (ACL) injury and reconstruction) who will receive a personalized training intervention; (ii) PROTO-PLX (PLX ​= ​placental derived mesenchymal like stromal cells) includes patients with moderate to advanced KOA (medial Osteoarthritis Research Society [OARSI] atlas joint space narrowing (JSN) grades 1 or 2) [40,41], in which cell therapy will be tested for safety, symptomatic and structural efficacy. A specific focus was on the assessment of synovitis [34,35], semi-quantitatively and quantitatively, without using contrast enhancement (CE). CE (using intravenous gadolinium administration) is considered the most accurate method to assess synovitis [34,35], as it permits differentiating synovitis as thickened, fluid-rich synovium that allows uptake of Gadolinium contrast molecules, but it is associated with scheduling challenges, higher cost, some patient burden, and potential medical risks [[42], [43], [44], [45], [46], [47]]. Furthermore, CE imaging requires monitoring by a physician, increasing cost and duration of the exam beyond actual scan time, hence advantages of assessing synovitis without its use.

A PubMed literature search was conducted for 2004 to 2024, with a specific focus on the past 5 years, using keywords relevant to MRI of KOA. No hard bibliometric measures were applied, but the subjective evaluation by the authors was used to rate the relevance, importance, and quality of the work with respect to the aims of this project. Relevance was evaluated with a focus on designing and implementing a KOA imaging protocol supporting the image assessments of interest within a short acquisition time, optimally considered less than 30 ​min. Many high-quality articles on the topic were therefore not included, as this is not a comprehensive literature review, but an expert opinion protocol development, with input from several OA and/or imaging experts (see acknowledgments). Their invitation was not contingent on a formal selection process, or on prescribing a formal structure to the interaction but on their direct or indirect involvement in the PROTO project. The following structural endpoints were deemed desirable for a KOA trial:

### 2.1 Quantitative

1) Cartilage thickness (ThC), volume (VC), surface areas (tAB, cAB), and denuded areas (dAB) [8,9,48].
2) Cartilage composition based on laminar (deep and superficial) T2;^10,30,49^
3) Hoffa synovitis (HS); [35,50], or synovitis at the intercondylar notch region [51].
4) Effusion synovitis (ES) [35,50].
5) Joint effusion, and synovial thickness, separately [34,35,52].
6) Meniscus morphology and position/extrusion [[17], [18], [19],^50^].
7) Bone parameters of size and shape, including osteophyte size [8,[20], [21], [22]].

### 2.2 Semi-quantitative

The MRI OA Knee Score (MOAKS) [3] or ACL OA Score (ACLOAS) [53]:

1) Cartilage damage/lesions;
2) Meniscus damage and extrusion;
3) Bone marrow lesions;
4) HS, potentially using a scoring system different from MOAKS [54] and different terminology of locations [51].
5) ES;
6) Joint effusion, and synovial thickening, separately;
7) Subchondral bone attrition;
8) Ligament status (cruciate and collateral), and loose bodies;
9) Bone pathology: i.e. subchondral sclerosis, subchondral insufficiency fractures, bone marrow infiltration, bone infection, acute and pathological bone fractures, and bone tumor infiltration.

Based on this selection, a KOA MRI protocol of net <30 ​min is suggested, supporting the above image assessments, limiting artifacts and imaging inconsistencies.

## 3 Results

### 3.1 Study duration, scanner hard- and software

Within PROTO, the MRI protocol will be acquired at baseline for cross-sectional analysis, longitudinal in the short term (6 or 12 months), and in the long term (36 months). If participants must terminate or withdraw from the study before 36 months, an “unscheduled” final MRI visit will be arranged, if possible. For KOA trials, MRI scanners with a field strength of ≥1.5 ​T (T) are recommended, but 3T is preferred. A multi-channel transmit-receive knee coil should be used, or a different type of coil of similar quality; surface/flex coils should be tested thoroughly before being included. Every attempt should be made to ensure that scanners and coils used in the study are not exchanged during the trial, even if with similar equipment from the same vendor. Although scanner breakdowns and repairs may occur, one should check during an upfront imaging site qualification process that no scheduled replacement takes place during the study. Software updates should also be avoided or kept to a minimum, as vendors do not normally disclose changes in their sequences between software versions. Hence, updates affecting fundamental scanner functions or the MRI sequences (e.g. new reconstruction algorithms) should be initiated before the start of the study, or delayed until the end of it, with the software version “frozen” during the study, to warrant consistent measurement and avoid systematic bias. If alterations in the hard- or software are indispensable, studying healthy volunteers before and after the change can help quantifying a potential bias, or assure that this is smaller than the expected effect of interest.

### 3.2 Suggested protocol, test-retest, and QC

**Fig. 1:**
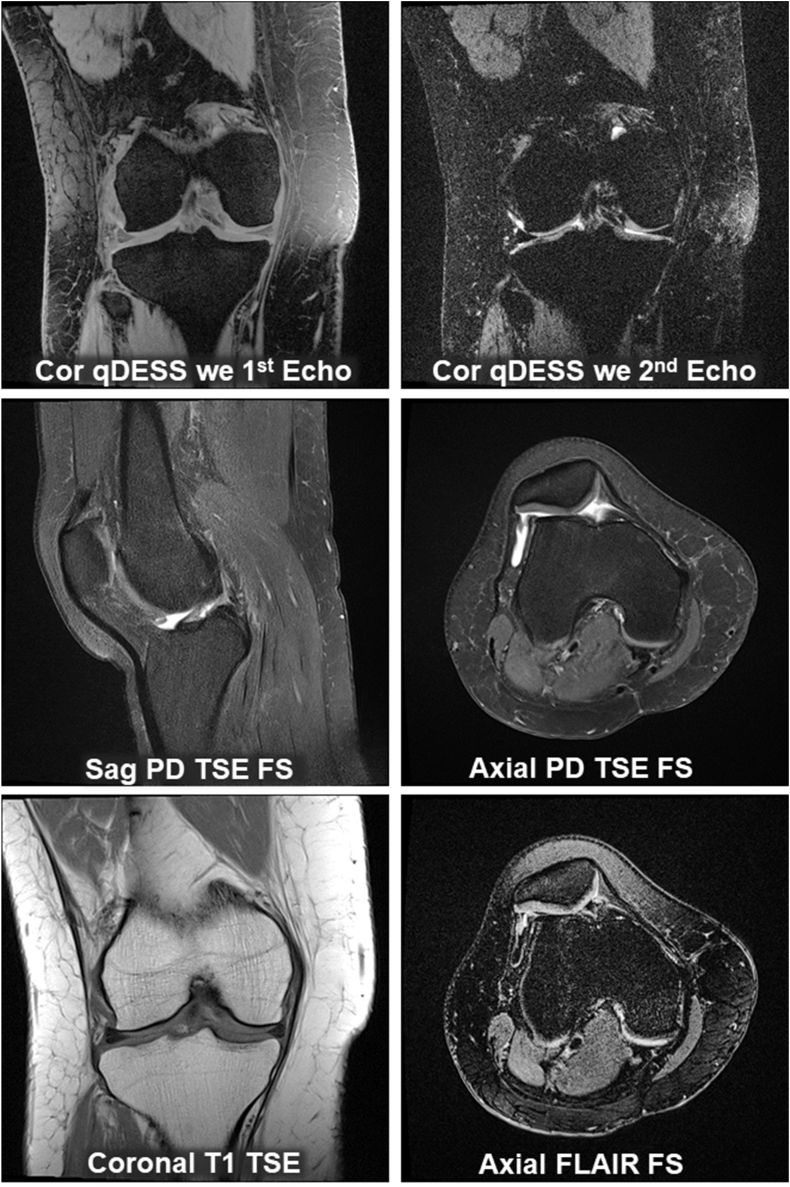
Example images of the proposed sample MRI acquisition protocol for clinical trials. These encompass the 1st and 2nd echo of the qDESS MRI sequence with water excitation (we). The 1st echo displays a mixed T1/T2 contrast, whereas the second one exhibits T2 and diffusion weighted imaging contrast. The “clinical” sequences in three orientations include a sagittal (sag) PD weighted TSE sequence with fat-saturation (FS) as well as an axially orientated PD TSE FS; the coronal (Cor) TSE sequence is acquired with T1 weighting (T1) and without FS. Finally, an axial FLAIR is obtained with FS. The MRIs were obtained on a 3T Siemens VIDA scanner (Software Version Numaris VA 50).

One knee per participant will be imaged (the “target” knee). The specific sequences facilitating semi-quantitative and quantitative image assessment within ≤30 ​min net imaging time are shown in Table 1 and Fig. 1, with detailed parameters in Table 2. The order of acquisition will be identical in each participant and visit.

**Table 1:** Summary of the MRI acquisition protocol, detailing the orientations, sequence acquisition durations, capabilities for semi-quantitative (sq) and quantitative (q) image assessments facilitated by the protocol. For sequence #2, two alternatives are provided (in grey).

	Orientation	MRI sequence	Acq. Time (minutes)	Assessments facilitated by the acquisition (sq or q) (assessment capability)
1A	3-Plane	Localizer(s)	0:14	
1B	Axial	Localizer	0:30	
2	Coronal	3D qDESS we 1.5/0.31 #	9:34	Cartilage thickness & T2 femorotibial joint (q); Meniscus morphology & extrusion (q); Bone size & shape (q)
2 alt. A	Sagittal same resolution	3D qDESS we 1.5/0.31 #	9:11	Cartilage thickness & T2 femorotibial & femoropatellar joint (q); Bone size & shape (q); Effusion synovitis (q); Hoffa synovitis (q)
2 alt. B	Sagittal near-iso-tropic resolution	3D qDESS we # 0.7/0.31 #	14:08	All of the above (2 and 2A)
3	Sagittal	2D PD TSE FS	2:13	Hoffa synovitis (q)§; Hoffa synovitis (sq) Hagiwara et al. (ref. #54) All tissues & pathologies MOAKS (sq); ACLOAS (sq)
4	Axial	2D PD TSE FS	3:56	Effusion synovitis (q), All tissues & pathologies MOAKS (sq); ACLOAS (sq)
5	Axial	2D FLAIR FS	6:38	Effusion & synovial thickness MOAKS (sq) Effusion & synovial thickness (q)§
6	Coronal	2D T1 TSE (no FS)	2:29	All tissues & pathologies MOAKS (sq), including medial and lateral collateral ligament status, loose bodies, subchondral sclerosis, subchondral insufficiency fractures, bone marrow & bone tumor infiltration, bone infection, acute and pathological bone fractures
Total acquisition time without re-test scan			25:34 ​min	

Acq. ​= ​acquisition; q ​= ​quantitative; sq ​= ​semi-quantitative; 2D ​= ​two-dimensional; 3D ​= ​three-dimensional; DESS ​= ​double echo steady state; we ​= ​water excitation (an alternative form of fat suppression); # ​= ​spatial resolution: slice thickness/in-plane resolution (both in mm); T2 ​= ​transverse relaxation time ​= ​compositional cartilage measure; alt. ​= ​alternative to 2 acquisition No 2; ref # ​= ​reference No; MOAKS ​= ​MRI Osteoarthritis Knee Score; ACLOAS ​= ​Anterior Cruciate Ligament Osteoarthritis Score; PD ​= ​proton density weighted; TSE ​= ​turbo spin echo; FS ​= ​fat suppression; § experimental and not yet established; FLAIR ​= ​fluid-attenuated inversion-recovery; T1 ​= ​T1-weighted.

**Table 2:** Detailed acquisition parameters of the proposed MRI acquisition protocol. For sequence #2, two alternatives are provided (in grey).

	Orientation	Sequence	FS	Acq. T	TR	TE	FA	Sl.Th.	Slice	FOV	Matrix	BandW	In-pl. reso.
				minutes	ms	ms		mm	No.	cm		Hz/Pixel	mm x mm
1A		Localizer	none	0:14	3.3	1.3	12	2.0	80	45	224 ​× ​224	540	2.01 ​× ​2.01
1B		Localizer HR	none	0:30	2630	83.0	15	3.5	15	16	192 ​× ​384	246	0.21 ​× ​0.21
2	Coronal	3D qDESS	we	9:34	16.9	4.9/29	15	1.5	80	16	512 ​× ​512	296	0.31 ​× ​0.31
2 alt. A	Sagittal	3D qDESS	we	9:11	16.9	4.9/29	15	1.5	80	16	512 ​× ​512	296	0.31 ​× ​0.31
2 alt. B	Sag near iso	3D qDESS	we	14:08∗	16.9	4.9/29	15	0.7	120	16	512 ​× ​512	296	0.31 ​× ​0.31
3	Sagittal	2D PD TSE	fs	2:13	3260	42	180	3.0	37	16	328 ​× ​448	248	0.36 ​× ​0.36
4	Axial	2D PD TSE	fs	3:56	4500	33	180	3.0	37	16	448 ​× ​448	248	0.36 ​× ​0.36
5	Axial	2D FLAIR	fs	6:38	9000	89	180	3.0	37	16	448 ​× ​448	219	0.36 ​× ​0.36
6	Coronal	2D T1-w TSE	none	2:29	600	9.9	180	3.0	37	14	307 ​× ​384	228	0.36 ​× ​0.36

FS ​= ​fat suppression; Acq. T ​= ​acquisition time; TR ​= ​repetition time; TE ​= ​echo time; FA ​= ​flip angle; Sl.Th. ​= ​slice thickness; No. ​= ​number; FOV ​= ​field of view; BandW = Bandwidth; Hz ​= ​Herz; In-pl. reso ​= ​in-plane resolution; sag near iso ​= ​sagittal near isotropic; for other abbreviations please see Table 1; ∗ the acquisition time is given to achieve the same signal-to-noise ratio as for the 1.5 ​mm sagittal qDESS we; f or sagittal and coronal acquisitions, a superior-inferior readout, and for axial acquisitions an anterior-posterior readout direction may be used to minimize aliasing artifacts.

A second (retest) scan not included in the 30-min net acquisition time) may be obtained during the baseline visit, if tolerated, with repositioning of the knee in the coil. The retest scan should be acquired for one sequence (acquisitions 2, 3, 4, or 5 in Table 1) per patient, assigned in ascending order, so that approximately an equal number of retest acquisitions is obtained for each sequence. A retest scan of the same imaging sequence will then again be acquired at 36 ​M (or any early termination visit). Acquiring test-retest scans at baseline and follow-up will permit evaluation of the precision (reproducibility) errors of quantitative image assessments and the smallest detectable change (SDC).

**Fig. 2:**
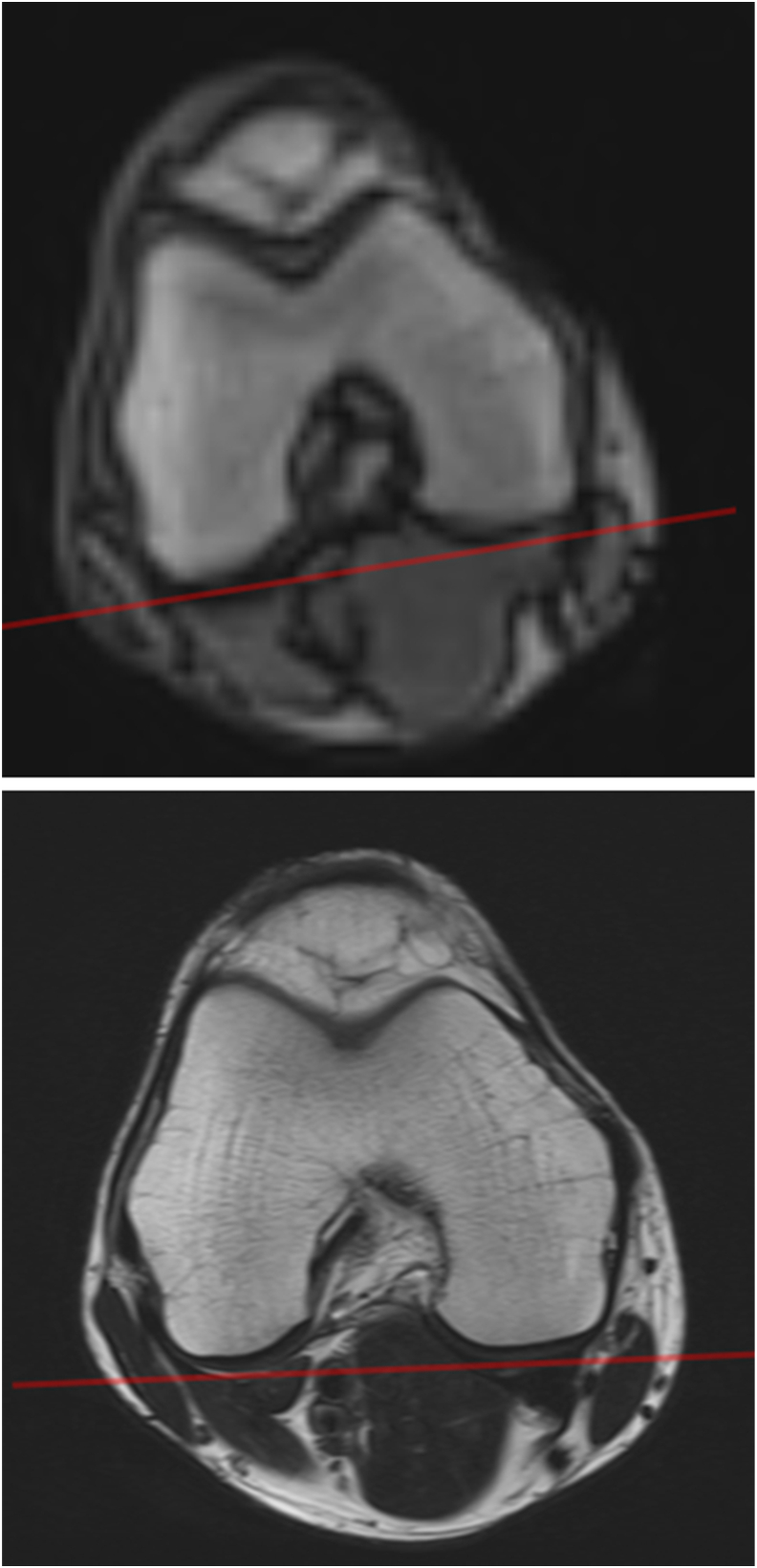
Standard (top) and high resolution (HR, bottom) axial localizer image for the double oblique coronal and sagittal acquisitions. Comparison of the localizer images demonstrates that the HR acquisition is superior in obtaining a double oblique coronal orientation (red line) pertinent to the DBEV, and in acquiring sagittal images perpendicular to the DBEV.

**Fig. 3:**
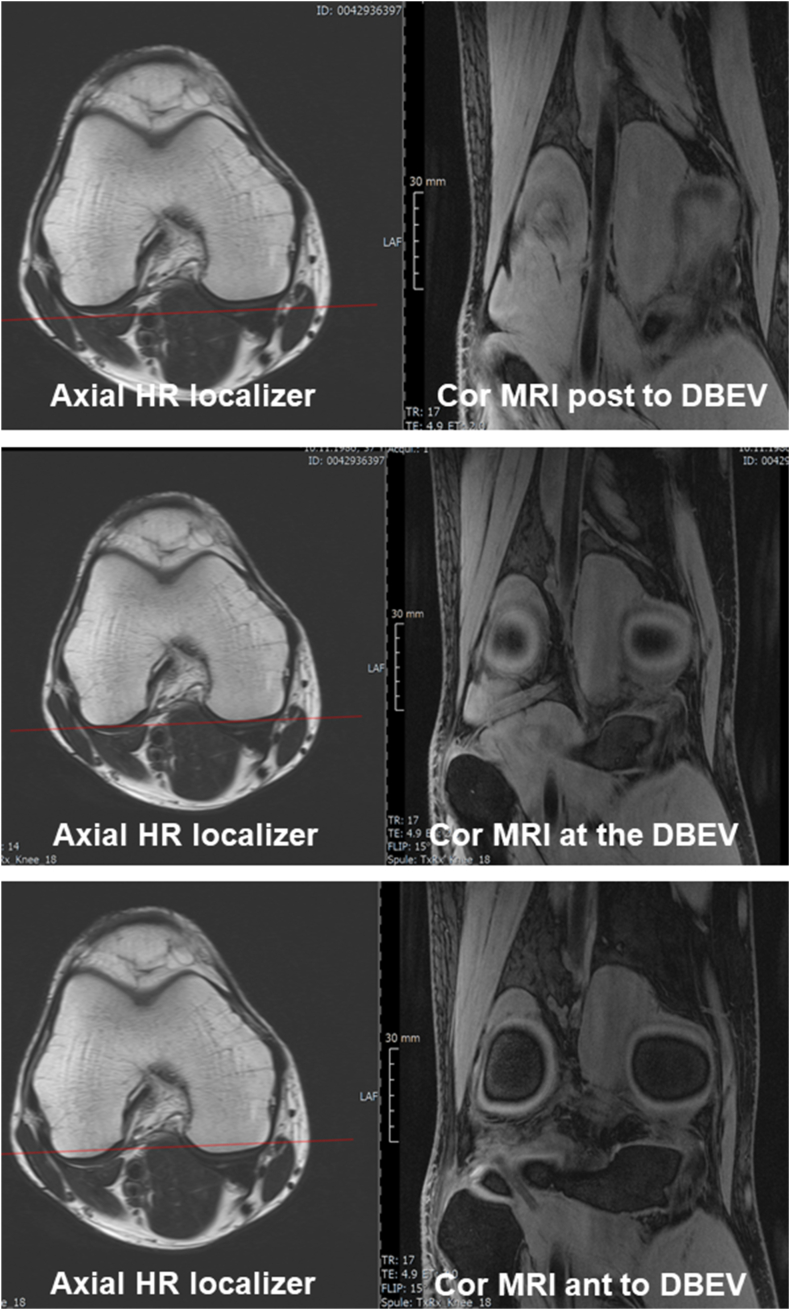
Axial HR localizer images shown on the left with a red line displaying the position of the respective coronal MRI. The middle image on the right shows a coronal MRI positioned double oblique to obtain a double bull eyes view (DBEV). The superior image on the right shows a coronal MRI posterior, and the inferior image a coronal MRI anterior to the DBEV. The MRIs were obtained on a 3T Siemens VIDA scanner.

To ensure high-quality data, each participant's exam must be quality controlled (QC'd) as soon as possible after the MRI session by a trained person familiar with the imaging acquisition guide and potential sources of errors/artifacts. In case of quality issues or non-adherence to the imaging protocol, the acquisition must be repeated. The QC review should include complete anatomical coverage, proper (double oblique) image orientation (Fig. 2, Fig. 3), proper fat saturation (FS) where applicable, lack of motion, aliasing (anatomical structures appearing at wrong locations) or other artifacts, and adherence to the pre-specified image acquisition parameters. If the parameters were not met and acquisition not repeated before the participant received drug/placebo treatment at baseline, all follow-up scans must be acquired with acquisition parameters identical to those of the baseline visit to ensure consistency of signal, contrast, and resolution, and warrant unbiased longitudinal measurement.

### 3.3 Patient preparation, positioning, and MRI scan orientations

Participants should avoid severe physical activity that involves knee loading over 12 ​h prior to imaging and be required to physically rest for 45 ​min prior to scanning. Participants should be positioned comfortably, supine in the scanner, with an empty bladder; sandbags and padding may be positioned around knee and ankle to fix the leg and minimize motion artifacts. The patella should be aligned with the middle of the coil, with the knee slightly flexed (10–15°). The leg may be placed in defined rotation, using positioning devices, but ensuring that the patient feels comfortable in natural external rotation, avoiding motion artifacts, we consider being more important. The position-sensitive magic angle effect is more pronounced in the femoral trochlea than in the weight-bearing femoro-tibial joint, and less relevant in longitudinal studies of cartilage T2, with “change over time” being the target measure.

The coronal, sagittal and axial acquisitions are planned with reference to the participant's knee position using a 3-plane localizer (scout), taking approximately 15 ​s (Fig. 2), that permits planning orientation and position of the actual MRI sequences (Table 1). The 3D localizer may be supplemented by a second, high-resolution axial localizer (Fig. 2) that depicts the posterior medial and lateral femoral condyles in better detail for proper orientation of the (double oblique) coronal and sagittal MRIs. For sagittal and coronal acquisitions, a superior-inferior readout, and for axial acquisitions an anterior-posterior readout direction may be used, as these minimize aliasing artifacts, which degrade image quality.

*Coronal* (acquisitions 2 and 6, Table 1): These sequences should be obtained as a double oblique, “double bull's eye view”. This view delineates the posterior medial and lateral femur in the same coronal image, and the condyle as circles of similar size (Fig. 2, Fig. 3). This approach allows for a consistent region of interest on the weight-bearing femur, ensuring that measurements are not affected by variable positioning, particularly in a longitudinal study. Using the axial localizer (Table 1, acquisition 1A), the slice showing the largest femoral cross-section is identified, and the second (high resolution) axial localizer (acquisition 1B) acquired in this position. A line connecting the posterior edges of both condyles is drawn, the coronal plane then orientated parallel to it (Fig. 2, Fig. 3). Based on the sagittal (localizer) image, the image slice is orientated perpendicular to the tibial plateau. Alternatively to the coronal orientation, a sagittal quantitative dual echo steady state (qDESS) may be acquired (Table 1, Table 2), using the same slice thickness proposed for coronal imaging, or with thin slices and near-isotropic spatial resolution (Table 1, Table 2). The field of view should be positioned to cover the femorotibial joint, to extend beyond the patella superiorly and beyond (approximately 0.5 ​cm) the margins of the soft tissues in medial-lateral direction to avoid “aliasing”.

*Sagittal* (acquisition 3, Table 1). This acquisition should be planned in a manner similar to the coronal ones. The axial scout is used to orient sagittal slices perpendicular to the line connecting the posterior femoral condyles, and based on the coronal localizer, perpendicular to the tibial plateau. The sagittal acquisitions should extend beyond the medial and lateral margins of the knee soft tissues to avoid aliasing, and the field of view anteriorly beyond the patella and posteriorly beyond the popliteal soft tissues. To assess synovitis, the position should extend about 5 ​cm above the patella, whereas the tibia needs to be included only to the level of the proximal tibiofibular joint, i.e. 2 ​cm distal to the plateau.

*Axial* (acquisitions 4 and 5, Table 1): These should be planned based on the coronal localizer with the largest diameter of the knee, and be angulated parallel to the tibial plateau. Based on the sagittal localizer, they should be oriented perpendicular to the posterior patellar surface. Because a focus of this protocol is assessment of ES and synovial thickness, the image stack should be placed more proximally than usual (see sagittal acquisition). This is important for comprehensively evaluating SE, but at the same time it is advised that the distal portion of the Hoffa's (or infrapatellar) fat pad (=IPFP) is covered for assessment of HS.

Table 1 summarizes which image assessments can be performed based on which MRI sequence at the expense of which acquisition time. This may be used as a “menu” to omit certain sequences and shorten the protocol, if tailoring to a specific study need is desired.

## 4 Discussion

The aim of this expert opinion design paper was to propose, implement, test, and discuss a state-of-the-art MRI acquisition protocol for KOA trials with <30 ​min net imaging time, enabling comprehensive and ideally automated [[36], [37], [38], [39]], quantitative image assessments. These included cartilage morphology and T2, synovitis, meniscus morphology and position, and bone size and shape. The protocol also supports semi-quantitative evaluation of structural tissue pathology, including bone disease [[3], [4], [5],^34^,^53^].

In clinical trials of KOA it is desirable to obtain short- (6–12 months) and long-term (36–60 months) imaging results. The former may demonstrate early structural effects and permit inclusion of participants who withdraw early, e.g. employing mixed-effects models [55]. Yet, long observation intervals are needed, acknowledging the slow progression rates in OA [56]. Tissue modification by pharmacological or non-pharmacological intervention likely requires a certain time to exceed thresholds above which they translate into symptomatic benefit and become clinically meaningful [57,58]. Particularly in longer trials, imaging centers must be directed to avoid scanner changes, because these may cause substantial deviations in quantitative measurement, even when from the same vendor [59]. Similarly, deviations occur when using different coils [60], so hardware changes are to be avoided. Participants should abstain from strenuous physical activity prior to imaging to avoid involuntary movement, compression of cartilage [[61], [62], [63], [64]], and modification of cartilage T2 [25,65,66].

OA trials of the early disease phase, when cartilage matrix is still intact, may prioritize assessment of cartilage composition [30], because no net cartilage loss occurs over prolonged periods of time [67]. However, subtle alterations in cartilage thickness may be detected in early OA models when specific, location-independent analysis techniques are used [68,69]. Furthermore, a semi-quantitative grading system has been proposed, specifically for ACL-injured patients with early structural changes [53]. Knee joint synovitis occurs at early KOA stages [70,71], including ACL injury [30]. It is associated with various imaging features [72]^,^ and considered a distinct “phenotype” of KOA [34,73,74], advocating its inclusion in clinical trials. In advanced disease, with radiographic JSN apparent, structural progression rates become greater and more stable, with cartilage thickness change apparent over relatively short periods of 6–12 months [75,76].

The core sequence of our proposed protocol is qDESS [[77], [78], [79]]. This sequence is not yet commonly available on MRI manufacturer software platforms, but can be installed by application specialists based on research agreements between the MRI manufacturer and the imaging site. This “research version” generates two separate images per repetition time, separated by a spoiler gradient that allows one to compute T2. The first acquisition displays T1/T2 contrast similar to proton density (PD) images, whereas the second acquisition exhibits T2 and diffusion-weighted contrast. Conventional DESS [15], on the other hand, only generates a single image with mixed T1-and T2-contrast [80], as root-sum-of-squares of the two images, permitting measurement of cartilage morphology, but not T2. When stored separately, however, a model-based voxel-by-voxel fit can produce a high-resolution T2 map [81]. Given a relatively long 2nd echo (2∗TR-TE; ∼30 ​ms) T2 can be estimated in both the deep and superficial cartilage layer [77]. MESE, for comparison, relies on approximate echoes, as it needs to sample non-T2 related effects, such as T1 and the flip angle, whereas with qDESS these can be modelled into the exponential fit so that two echoes suffice [[77], [78], [79]]. QDESS T2 was shown to match MESE T2 [77] and single echo spin echo T2, considered the gold standard [82], and to discriminate between mild vs advanced KOA [83]. With qDESS, T2 and cartilage morphology can be analyzed from the same high-resolution images, obtained with relatively short imaging time, without requiring separate segmentations, and substantially reducing analysis and patient burden [77]. The coronal orientation of the qDESS acquisition (parallel imaging, readout superior-inferior, phase encoding right-left) takes <10min, whereas SPGR (for cartilage thickness) and MESE (for T2) taken together take much longer [80]. Even shorter qDESS imaging times <5 ​min have been published [77], but the current protocol focusses on high in-plane resolution (0.31 ​mm) for accurate cartilage thickness analysis. Although such resolution may not be required for measuring bulk T2, but it is advantageous for separating T2 for in the deep and superficial layers, in which compositional properties differ substantially [49,84]. The coronal (and standard sagittal) slice thickness proposed here (1.5 ​mm) is geared towards a greater signal and contrast-to-noise ratio compared with the near-isotropic OAI protocol [80] (0.7 ​mm), with sensitivity to longitudinal cartilage thickness changes shown to be similar when analyzing every second vs each 0.7 ​mm slice [85]. The sequence proposed here is geared towards the greater signal and contrast-to-noise ratio associated with greater slice thickness, whereas isotropic or near-isotropic acquisition can be more easily reformatted to other planes, permitting assessment in all image orientations (see Table 1: 2Alt B). Near-isotropic imaging typically occurs at the expense of a lower in-plane resolution [77], which is disadvantageous when measuring thickness (or laminar T2) of a thin (sometimes denuded) cartilage layer, or comes with the expense of a longer acquisition time. Partial volume effects of femorotibial cartilage are smaller with coronal than with sagittal orientation, with a well-defined and consistent orientation of the double oblique acquisitions warranting consistent and reproducible measurements [86,87]. A coronal acquisition is also beneficial when measuring meniscus extrusion [17,18], with automated methods being developed [50]. Osteophyte measurement at medial or lateral locations also require coronal acquisitions, with a coronal multi-planar reconstruction of a sagittal near-isotropic qDESS as fall back, also applicable for meniscus extrusion [19,88]. If the patellofemoral joint is to be included, a sagittal protocol is mandatory [89] and is advantageous for analysis of bone size and shape [8,[20], [21], [22]] (except for measuring medial and lateral osteophytes). Sagittal qDESS also may be used for evaluating HS and ES [51,90], potentially relying on axial multiplanar reconstruction if acquired with near-isotropic resolution.

With conventional DESS [15,80], fully automated cartilage thickness measurement was achieved using deep learning and convolutional neural networks methods, validated cross-sectionally [91,92] and longitudinally [93]. Fully automated laminar T2 analysis from qDESS hence is straightforward based on validated technology [[91], [92], [93]].

The acquisition time for the three clinical turbo spin echo (TSE) sequences images is ​< ​9min. Recent innovation has reduced acquisition time to approximately 5min, using combined simultaneous multi-slice and parallel imaging acceleration for multi-contrast knee MRI [94], or qDESS with deep learning super-resolution augmentation [95]. Deep learning-based acquisition and image reconstruction may accelerate acquisition of clinical TSE sequences 10-fold, with 2–3 ​min being sufficient for the exam [96,97], and 5–7 ​min saved with the present protocol.

PD TSE FS sequences are fluid-sensitive, but they cannot discriminate between the actual (potentially thickened) synovium and joint effusion because both pathologies appear hyper-intense. However, CE imaging, at one time point after gadolinium injection, and particularly “dynamically” at several time points (DCE-MRI) permit differentiation between the (thickened) synovial membrane and effusion [34], synovial thickening representing the most accepted MRI measure of synovitis [35]. In end-stage OA, DCE-MRI was found to be strongly associated with synovitis histology [98]. Whereas static CE-MRI added significant independent information to this association, non-CE-MRI (NCE-MRI) did not [98]. Given the inability of a standard fluid-sensitive sequence to differentiate between effusion and synovial thickening, “ES” has been used as a surrogate for synovitis [3,34]. Likewise, signal heterogeneities in the IPFP or inter-condylar region (HS) [51] have been proposed as surrogates of ES, with evidence that the IPFP plays a role in KOA symptomatology [99]. IPFP signal heterogeneity was shown to be related to synovitis on biopsy, and to synovitis detected on CE T1-weighted TSE MRI [71]. Perfusion variables on DCE MRI of HS were more strongly associated with knee pain severity than MOAKS HS, suggesting inflammation in the IPFP to be associated with knee pain [100]. Yet signal characteristics from the IPFP on NCE-images are non-specific when acquired without CE [34], whereas the latter increases patient burden [[42], [43], [44], [45], [46], [47]]. A specific reading system has been proposed for superior semi-quantitative evaluation of HS [54]. Segmentation and quantification of the IPFP [51] has been shown to be reproducible [101] and responsive to change in weight [102,103]. Furthermore, a correlation of IPFP MRI signal has been demonstrated with KOA status and progression [[104], [105], [106]]. Finally, IPFP signal alterations were shown to be related to synovial fluid cytokine profiles [107] and to higher serum levels of resistin, an adipose-derived hormone and endogenous ligand of Toll-like receptor-4, triggering major inflammatory pathways and mediating inflammatory processes [108]. Although HS has thus far been mainly graded semi-quantitatively [3,34,54,109], efforts are underway to obtain quantitative HS measures [34,50]. Extracting HS measures quantitatively may feed statistical approaches with continuous variables to improve diagnostic accuracy longitudinal sensitivity to change [50]. QDESS also has been explored for semi-quantitative scoring of HS in the intercondylar region, the agreement vs CE-MRI of synovial thickness [51] in this region being similar to other regions around the knee.

Whereas sagittal PD TSE is typically used to assess HS, axial PD TSE is more tailored towards assessing ES. Imaging should reach more proximal for this purpose, aiming at full coverage of effusion volume, with proximal regions being the most common locus [110]. Semi-quantitative evaluation of ES on NCE-MRI was shown to be positively correlated with quantitative measures of synovial thickening and effusion on CE-MRI, this relationship being stronger than that for HS with CE-MRI [35]. Biochemical serum markers of inflammation (i.e. sHA and sMMP-3) were modestly associated with ES; clinical signs of effusion were not highly sensitive but highly specific to presence of ES on NCE-MRI. However, in at least one study, HS (on NCE-MRI) was not associated with ES [111]. Several investigators have taken quantitative approaches to measure ES (mostly maximal area or volume) on NCE or CE TSE [52,[112], [113], [114], [115]]. ES volume was shown to be statistically significantly related to semi-quantitative scores of ES [3,4,34,116]. Although volumetric measurement on NCE PD TSE FS overestimated the amount of ES from T1-weighted CE FS imaging (as a reference) almost two-fold, both measurements were highly correlated [114]. A quantitative approach was applied in a clinical trial reporting volumetric ES to be reproducible in participants with an inflammatory OA phenotype [117]. Studies investigating ES pathophysiology using novel automated methods are underway [50].

Given the inability of classical fluid-sensitive NCE imaging to differentiate between synovial inflammation and effusion, there are efforts in developing novel NCE sequences that measure “synovitis” (with synovial thickening) directly, rather than the surrogate composed of effusion and synovium together. QDESS is one of these sequences, since a weighted subtraction of the two qDESS echoes can null the synovial fluid and create a positive contrast to visualize synovium [90]. Although qDESS was shown to underestimate semi-quantitative synovitis scores compared with CE-MRI, the two methods were highly correlated [90]. Diagnostic confidence was slightly superior for qDESS, although inferior to that of CE-MRI [51]. The attractiveness of the qDESS clearly lies in its multi-functionality of supporting quantitative endpoints in KOA. Another option for evaluating the synovium without CE is double inversion recovery MRI [118,119]; however, this has not been explored much and requires further validation.

We further selected an axial fluid attenuated inversion-recovery (FLAIR) sequence with FS, with acquisition parameters at 3T having been explored [33]. Compared with PD TSE, the acquisition time is relatively long due to the need for nulling the fluid signal [120] and the inversion time is longer (2200 ​ms) than that commonly used in brain imaging [33]. Before a 90° excitation radiofrequency pulse is applied with FLAIR, a 180° inversion pulse is administered and a saturation pulse is utilized for chemical shift-selective FS. The FLAIR FS improves delineation of the synovium in the intra-articular space by suppressing both the fluid and fat signal. The absence of the fluid signal increases the dynamic range and improves delineation of other tissues such as synovium [120]. The FLAIR sequence is acquired in identical location, orientation, and resolution as the axial PD TSE FS. Given improved conspicuity of the synovium and the superior separation between thickened synovial membrane and synovial fluid (without CE), FLAIR has shown superior diagnostic performance to standard clinical PD TSE sequences in relation to CE T1-weighted SE FS as a reference standard [33]. If effusion volume is to be measured, axial acquisitions should extend 5 ​cm above the patella, particularly when evaluating longitudinal change over time. An “accelerated” FLAIR [120] (at 3T) has been introduced with acquisition times of approximately 50% less, similar to that of axial PD TSE FS. The accelerated FLAIR has shown equivalence to CE T1-weighted SE FS in terms of intra-reader and inter-reader reproducibility, and the diagnosis of inflammatory knee synovitis [120]. Neither qDESS or FLAIR have yet been explored in terms of quantitative assessment of HS, ES, or synovial thickness.

Contrary to other clinical protocols, the proposed coronal TSE is acquired T1-weighted and without FS. This better suits for evaluation of the collateral ligament complexes and bone pathology, e.g. subchondral bone sclerosis, subchondral insufficiency fractures, bone marrow infiltration, bone infection, acute and pathological fractures, bone tumor infiltration, and other pathologies, important to monitor or exclude from KOA clinical trials.

Repeat (test-retest) scanning for each sequence is useful from a clinical research perspective, permitting quantification of the test-retest precision (root mean square standard deviation and coefficient of variation) ≤and specific to the study [121]. Furthermore, if performed at two longitudinal time points, repeat scanning allows for determining the smallest detectable change (SDC), relating test-retest precision to the actual longitudinal change within the study [[122], [123], [124]]. This provides thresholds of progression/non-progression and enables transformation of continuous data into number of progressors per treatment and placebo arm, a metric often more straightforward to communicate to regulators or the public. Ideally, retest scanning should be performed at another day, to include variability originating from scanner drift, imaging conditions, and patient conditions. However, longer term (8–9 month) measurement variability in cartilage morphology only is marginally greater than that within the same imaging session (with repositioning of the joint), the relative position of the images vs the joint anatomy apparently being the major driver of test-retest error [125]. It is of utmost importance that all MRIs are thoroughly checked as soon as possible after the session. In case of quality issues, any unacceptable acquisitions should be repeated, ideally while the patient is still on site. This must occur before the patient is receiving drug or placebo treatment.

Limitations of the current protocol include the lack of inclusion of deep learning-assisted clinical sequences that may save 5–7 ​min over the “clinical” TSE [96,97], but these were not available on the VIDA scanner used for the PROTO studies. The qDESS currently has “research” status, and inclusion in a clinical trial requires administrative and technical work between the MRI site, application specialists, and the MRI manufacturer. While T2 may be sensitive to proteoglycan content [23,126], other MRI techniques such as T1rho, sodium imaging, or gagCEST, are more specific to these or other matrix components [10,23,32,127]. Such sequence hence should to be added if these measures are of particular interest. In the current protocol, no gold standard CE-MRI is obtained for assessing synovitis. For logistical and safety reasons [[42], [43], [44], [45], [46], [47]], use of CE appears feasible in dedicated centers for early phase trials, but measurement from NCE-MRI (even if only surrogates) is often preferred from a logistic perspective. We decided to include the NCE FLAIR [33], with the accelerated version [120] not available on the scanner. To reduce whole study imaging time, FLAIR of course may be omitted if acquisition time is limited and synovitis is not a focus of the study.

In conclusion, this work is intended to bridge the gap between common technical manuals for image acqusitions in clinical studies and the overwhelming amount of imaging literature. In this expert opinion clinical study protocol design paper, we propose an MRI acquision protocol for KOA trials suitable for early and advanced KOA. The protocol aims to balance imaging efficiency (<30min), safety (no intravenous 10.13039/100021407CE), and academic interest, with a comprehensive number of relevant semi-quantitative and quantitative imaging endpoints supported. These are pertinent to the multitude of articular tissues and pathological processes, including synovitis. The availability of a relatively short and efficient image acquisition protocol, supporting relevant semi-quantitative and quantitative imaging endpoints with the potential for automated analysis, is instrumental for conduct of clinical research. The protocol should provide sufficient opportunity for technical innovation and for setting up innovative clinical trials on pharmacological, non-pharmacological, “advanced therapy medicinal product”, surgical, or other interventions, either for improving knee tissue status or for ameliorating articular and peri-articular structural pathology. Once an effective disease-modifying therapy is established, abbreviated protocols may be used to monitor the structural efficacy of such interventions in the long term [58].

## Consent for publication

All authors have consented to publication.

## Authors’ contributions

Study conception and design: FE, TCWR, WW, TW.

Acquisition of data: TCWR.

Analysis & interpretation of data: all authors.

Writing of the first manuscript draft: FE.

Critical manuscript revision and approval of final manuscript: all authors.

## Role of the funding source

This work was funded by the European Union under Grant Agreement Nr. 101095635 (PROTO – Horizon Europe). Views and opinions expressed are however those of the authors only and do not necessarily reflect those of the European Union or the European Health and Digital Executive Agency. Neither the European Union nor the granting authority can be held responsible for them. The design of the image acquisition protocol and the content of this article has not been contingent upon approval from the study sponsor.

## Ethics approval

Ethics approval of the two PROTO clinical studies is pending.

## Declaration of competing interest

FE is CEO/CMO and co-owner of Chondrometrics GmbH; has provided consulting services to Merck KGaA, Kolon-Tissuegene, Servier, Galapagos, Novartis, 4P Pharma/4Moving and Trialspark/Formation Bio; and has – related to this paper – received funding through PROTO from the EU.

TCWR has no conflict of interest to declare

AC has received research support from the National Institutes of Health, GE Healthcare and Philips and has provided consulting services to Patient Square Capital and Elucid Bioimaging Inc – unrelated to this paper.

NMB has – related to this paper – received funding through PROTO from the EU

TM has -– related to this paper – received funding through PROTO from the EU

GND has – related to this paper – received funding through PROTO from the EU

AW is a part-time employee of Chondrometrics GmbH and has – related to this paper – received funding through PROTO from the EU

WW is part-time employee and share-holder of Chondrometrics GmbH and has – related to this paper – received funding through PROTO from the EU

TW is part of the Executive Board of the Advanced Therapies in Orthopaedics Foundation and has – related to this paper– received funding through PROTO from the EU.
